# Recent advances in modulation of cardiovascular diseases by the gut microbiota

**DOI:** 10.1038/s41371-022-00698-6

**Published:** 2022-04-25

**Authors:** Sepiso K. Masenga, Benson Hamooya, Joy Hangoma, Valerie Hayumbu, Lale A. Ertuglu, Jeanne Ishimwe, Sharla Rahman, Mohammad Saleem, Cheryl L. Laffer, Fernando Elijovich, Annet Kirabo

**Affiliations:** 1 Mulungushi University, School of Medicine and Health Sciences, HAND Research Group, Livingstone, Zambia; 2 Vanderbilt University Medical Center, Department of Medicine, Nashville, TN USA

**Keywords:** Physiology, Cardiovascular diseases

## Abstract

The gut microbiota has recently gained attention due to its association with cardiovascular health, cancers, gastrointestinal disorders, and non-communicable diseases. One critical question is how the composition of the microbiota contributes to cardiovascular diseases (CVDs). Insightful reviews on the gut microbiota, its metabolites and the mechanisms that underlie its contribution to CVD are limited. Hence, the aim of this review was to describe linkages between the composition of the microbiota and CVD, CVD risk factors such as hypertension, diet, ageing, and sex differences. We have also highlighted potential therapies for improving the composition of the gut microbiota, which may result in better cardiovascular health.

## Introduction

Humans are surrounded, both externally and internally, by a diverse range of microbes which profoundly affect wellbeing by interacting with skin, respiratory, and digestive systems. They self-organize, quickly mold to their changing environment and develop a complex ecosystem within an otherwise uninhabitable niche. The human halobiont is a very diverse assembly of microbial species which makes a singular functional unit [1]. The gastrointestinal tract harbors a complex community of over 100 trillion microbial cells that influence human physiology, metabolism, nutrition, and immune function. Therefore, the gut microbiota is considered a singular functional unit sometimes termed ‘metabolic organ’ [2]. Some research estimates suggest that human gut possesses ~1000 bacterial species with 100-fold more genes than those found in the human genome [3, 4].

**Fig. 1:**
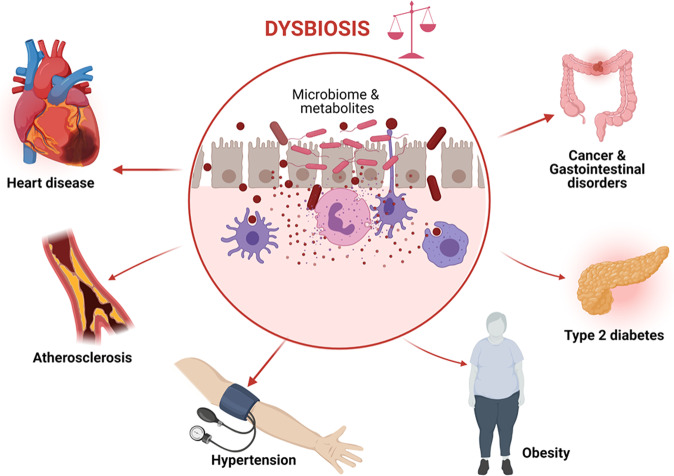
Diseases associated with dysbiosis. Abnormal changes in the composition of the microbiota (dysbiosis) is positively associated with pathogenesis and propagation of heart disease, atherosclerosis, hypertension, obesity, type 2 diabetes mellitus, cancer, and gastrointestinal disorders.

The gut microbiota can exert healthy benefits as well as pathological effects on human health [5, 6]. Physiological functions of the microbiota include metabolism of food, fermentation of indigestible food, synthesis of vitamins, and forming an epithelial barrier and barricade against pathogenic bacteria [7]. Dysbiosis, a term referring to changes in the composition of the microbiota and its metabolites has been suggested to play a pivotal role in propagating inflammatory and metabolic diseases including gastrointestinal disorders, cancers, cardiovascular disease (CVD) [6], atherosclerosis, hypertension, kidney disease, heart disease, obesity, type 2 diabetes mellitus, and inflammatory bowel disease (Fig. 1) [5, 8]. Several metabolic pathways may mediate the pathogenic effects of an altered microbiota, including the trimethylamine (TMA)/trimethylamine N-oxide (TMAO) and the bile acids pathways [8]. TMAOs have been associated with increased risk for CVD [9]. In the last decade, the relationship between the microbiota and cardiovascular disease has become a major topic of interest. This review presents a detailed and comprehensive overview of the published literature in the last decade regarding some of the mechanisms, recent advances, diagnostic approaches, and clinical implications of the gut microbiota in contributing towards CVD.

## Gut microbiota, atherosclerosis and CVDs

**Fig. 2:**
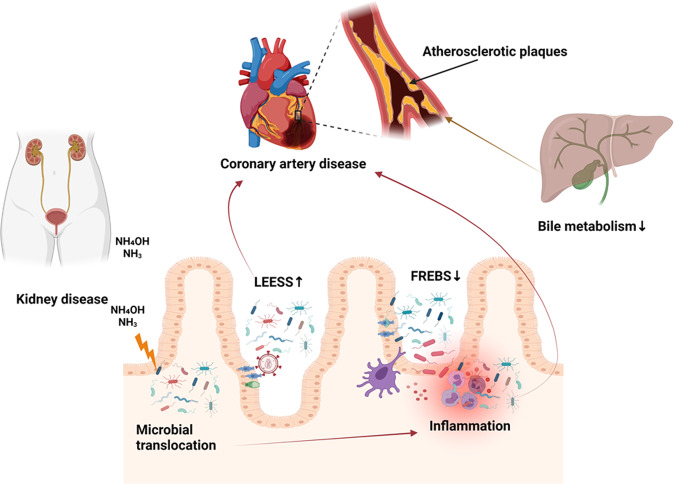
Microbiota’s contribution to atherosclerosis and CVD. Ammonia (NH_3_) and ammonium hydroxide (NH_4_OH) resulting from kidney disease or the action of microbial urease and HIV infection in the gut contributes to microbial translocation and systemic inflammation. Microbes colonize atherosclerotic plaques enhancing progression of various atherosclerotic processes. Dysbiosis contributes to decreased bile formation that results in decreased cholesterol elimination and increased plasma levels of low-density lipoproteins. LEESE *Lactobacillus, Esherichia, Enterococcus, Shigella,* and *Streptococcus*, FREBS *Faecalibacterium, Roseburia, Eubacterium rectale, Bacteroides fragilis*, and *Subdoligranulum*.

The source of many of the microorganisms that have been associated with atherosclerotic plaques, endothelial dysfunction, and resulting CVDs is their translocation from the gut into the systemic circulation. Metabolites produced by the microbiota may also promote kidney injury as they are concentrated and excreted in the kidney [10]. Conditions that increase microbial translocation from the gut, such as HIV infection, overproduction of TMAOs and urea have been linked to systemic inflammation, heart failure, and hypertension [11]. Microbial urease leads to overproduction of waste products, such as ammonia and ammonium hydroxide, which are especially important in patients with chronic kidney disease (CKD), whose urea excretion is already compromised [12]. Overproduction of ammonia and ammonium hydroxide disrupt the tight junctions between intestinal epithelial cells resulting in further enhancement of microbial translocation and systemic inflammation [8, 9]. While the precise mechanism by which the microbiota contributes to atherosclerosis remains unknown, dysbiosis has been consistently associated with a leaky gut, with abnormalities of lipid and glucose metabolism that are associated with inflammation, and with the size of atherosclerotic plaques, which ultimately contribute to the development and progression of CVD and to its prognosis [13]. It has been shown that atheromatous plaques of patients with coronary artery disease (CAD) contain pathogenic *Staphylococcus* species, *Proteus vulgaris, Klebsiella pneumoniae*, and *Streptococcus* species [7]. Their guts exhibit an increase in *Lactobacillus, Streptococcus, Esherichia, Shigella* and *Enterococcus* species, concomitant with a reduction in *Faecalibacterium, Subdoligranulum, Roseburia, Eubacterium rectale* and *Bacteroides fragilis* species, the latter group known to regulate T-cell functions in the gut mucosa with consequent anti-inflammatory effects and protection of the gut barrier [7, 14]. In patients at high risk for stroke, there is a reduction in butyrate-producing bacteria such as those of the *Lachnospiraceae* and *Ruminococcaceae* family, resulting in reduced fecal butyrate levels and concomitant increases in intestinal pathogens such as those of the *Enterobacteriaceae* and *Veillonellaceae* family [7]. Whether microbiota has a direct role in the pathogenesis of other CVDs such as abdominal aortic aneurysm (AAA) or peripheral artery disease (PAD) is yet unknown but likely, since they contribute to inflammatory processes and colonization of atheromatic plaques in blood vessels, thereby enhancing the progression of various atherosclerotic processes (Fig. 2). More studies are required to understand the mechanisms and so devise future therapeutic interventions. For example, reduced bile acid synthesis by a dysbiotic microbiota has been shown to decrease the amount of cholesterol eliminated via feces, with increases in absorption and plasma levels of low-density lipoproteins. This may be an additional mechanism that contributes to increased risk for atherosclerosis and CVD in subjects with a dysbiosis [7, 9, 15].

### Gut microbiota and hypertension

The pathophysiology of hypertension involves various contributing factors including genetic, lifestyle, environmental, hormonal, inflammatory, and hemodynamic changes. Mounting evidence from human and animal studies suggests that gut microbiota play an indispensable function in the regulation of blood pressure [16–29]. The evidence for an association between gut microbiota and hypertension emanates from studies in murine models showing that rats lacking normal gut flora experience elevated blood pressure [29, 30]. Moreover, alterations in the composition of fecal microbiota have been linked to modulation of blood pressure and poor response to antihypertensive drugs [8]. Alpha diversity is the parameter that reflects microbial diversity within a particular ecosystem, as captured in a biological sample. Reduced alpha diversity of the microbiota has been identified in hypertensive patients. [17–25, 28]. Similar trends were observed in obesity, hyperinsulinemia, and dyslipidemia. Moreover, studies in humans demonstrated an association between a higher abundance of Gram-negative microbiota including *Klebsiella*, *Parabacteroides, Desulfovibrio*, and *Prevotella* and higher blood pressure levels, but not all studies confirmed this pattern [16, 18, 21, 26]. The cross-sectional HELIUS cohort study (HEalthy Life In an Urban Setting study) demonstrated positive correlations between *Klebsiella spp*. and *Streptococcaceae spp*. and blood pressure [24], and confirmed the results from previous studies [25, 26]. A causal relationship is suggested by experiments with fecal microbiota transplantation (FMT). It was clearly shown that germ-free (GF) mice, which received FMT from a hypertensive patient not only developed a similar gut microbiota as that of the donor, but also elevated systolic and diastolic blood pressures after 8 weeks when compared with GF mice that received FMT from normotensive donors [22]. Also, stroke-prone SHRs (spontaneously hypertensive rats) harbor a dysbiotic gut microbiota that differs significantly from that of normotensive WKY (Wistar-Kyoto) control rats. FMT from SHRs into WKY controls increased the systolic blood pressure of these otherwise normotensive rats [29]. Additional studies in Dahl salt-sensitive rats [31], angiotensin II infused mice [32], high salt treated mice [17], and deoxycorticosterone acetate-salt hypertensive mice [33] demonstrated that all these hypertensive animal models exhibit dysbiosis. Santisteban et al. recently showed that SHRs exhibit the pathophysiological changes and disrupted integrity of the gut epithelium, characteristic of other forms of dysbiosis [34]. Finally, it has been shown that abnormal intestinal permeability and dysbiosis can be reversed by treatment with the antihypertensive agent losartan [35], suggesting that the relationship between dysbiosis and blood pressure may be bidirectional.

Several studies have implicated high salt in contributing to the dysbiosis of both human and experimental animals. A seminal study from Muller and colleagues demonstrated that high salt treatment depleted *Lactobacillus murinus* from the gut microbiota, resulting in an increase in TH 17 cells and salt-sensitive hypertension, findings that were replicated in a pilot study in humans [17]. Since high salt depleted *Lactobacillus spp*. and raised blood pressure both in human and animals, this study indicates that the link between gut microbiota and hypertension is not species-specific. Interestingly, other studies demonstrated that either reduced salt or increasing *Lactobacillus spp* with probiotic treatment improved blood pressure regulation, arterial compliance, vascular function, and insulin sensitivity [17, 36, 37]. An elegant systematic review and meta-analysis of randomized, controlled trials showed that probiotics containing *Lactobacillus spp* are effective in blood pressure regulation if used in sufficient amount for at least 8 weeks [38].

**Fig. 3:**
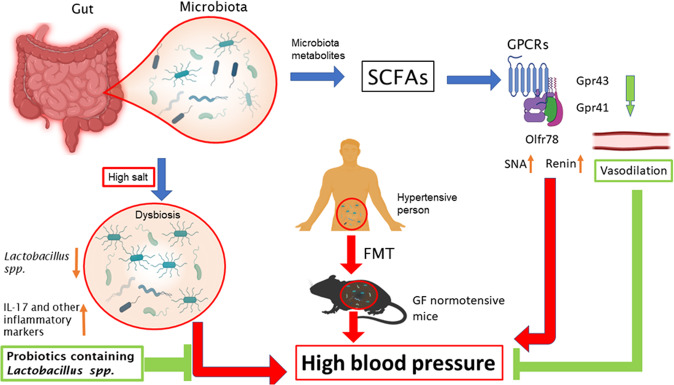
Gut microbiota and high blood pressure. Microbiota metabolites SCFAs modulate distinct GPCRs and thereby affect blood pressure. For example, activation of Gpr43 and 41 results in vasodilation and blood pressure attenuation. In contrast, activation of olfr78 increases SNA and renin secretion resulting in blood pressure elevation. Moreover, high salt depletes lactobacillus spp. causing dysbiosis and activation of inflammatory immune response by releasing IL-17 and other inflammatory signaling molecules consequently causing blood pressure elevation. FMT is strong evidence to show that gut microbiota plays an indispensable role in the contribution of high blood pressure. SCFAs short-chain fatty acids, GPCRs G protein-coupled receptors, SNA sympathetic nerve activity, FMT fecal microbiota transplantation, GF germ free.

Short-chain fatty acids (SCFAs) resulting from microbiota metabolism have been linked to blood pressure mediated by G-protein coupled receptor (GPCR) pathways in renin secretion and blood pressure regulation [39]. Olfactory receptor (Olfr) 78 and GPR41 free fatty acid receptor stimulation by SCFA results in elevated and decreased BP, respectively [8]. SCFAs such as acetate and propionate produced by gut microbiota have antihypertensive effects by decreasing systemic inflammation and atherosclerotic lesions which are independent predictors of hypertension [39]. A composition of the microbiota characterized by abundant *Lactobacilli* is known to have BP lowering effects. Other SCFA produced by the gut microbiota such as lactate and butyrate also have a significant impact on BP through vasodilation and vasoconstriction mediated by GPR43, GPR41, and Olfr 78 [39]. A summary of the relationship between the gut microbiota and blood pressure is illustrated in Fig. 3.

Although research data indicate a great potential to target the gut microbiota in contributing to treatment of hypertension by using probiotics, changing lifestyle, and diet, further research is warranted to better understand the role of various gut microbial species and their metabolites in the regulation of blood pressure and associated diseases. Further, it would be interesting to understand the interaction among environmental factors, gut microbial species, and blood pressure regulation.

### Dietary lifestyle, microbiota, and CVD

**Fig. 4:**
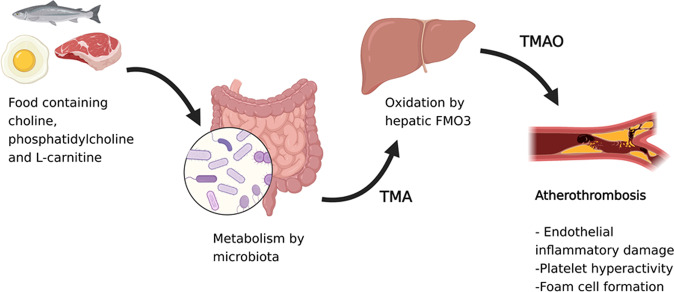
TMAO biosynthesis and metabolism. Choline, phosphatidylcholine, and l-carnitine found in fish, red meat and eggs are metabolized into TMA by colonic microbiota. The TMA that enters the systemic circulation is oxidized into TMAO by FMO3 in the liver, which is released back into the circulation, leading to platelet and inflammatory pathway activation. Inflammatory injury in the endothelium, along with increased foam cell formation and platelet activation, contributes to the progression of atherosclerosis and development of atherothrombotic events.

Evidence suggest that diet potentially modulates the gut microbiota by regulating the balance between pathogenic and beneficial microbes or microbial products [40]. Vegetarian diets foster a beneficial microbiota composition by increasing *Prevotella* enterotype whereas diets high in animal protein foster *Bacteroides* enterotype and other species associated with proatherogenic metabolites and CVD [40, 41]. The production of the proatherogenic metabolite TMAO, resulting from TMA oxidation by the liver enzyme flavin monooxygenase 3 and its release into the systemic circulation have been linked to coronary plaques, peripheral artery disease, the severity of CVD, and its complications including stroke, myocardial infarction, and death [42, 43]. Common dietary nutrients possessing a TMA moiety, such as the choline, phosphatidylcholine, and l-carnitine present in red meat, fish, and eggs after microbial metabolism, are the main contributors of TMAO-mediated effects that promote artherosclerosis [9]. The underlying mechanisms by which TMAO contributes to CVD remain unknown. However, preliminary evidence suggests that TMAO stimulates inflammatory pathways with activation of cells of the innate immunity response that propagate atherosclerosis. Also TMAO interferes with platelet function through stimulus-dependent calcium signaling, promoting atherothrombotic events (Fig. 4) [44].

Diets rich in fiber such as whole grains increase the acetate-producing *Bifidobacteriaceae*, which are protective against pathogenic bacteria, lower blood pressure, improve insulin sensitivity, and decrease cardiac hypertrophy and fibrosis [8, 45]. Polyphenols, a large class of aromatic compounds found in plant-based beverages have been shown to improve cardiovascular health through their antiplatelet and anti-inflammatory actions, and by inducing nitric oxide formation in blood vessels, promoting vasodilation and improving gut microbiota with increased *Firmicutes* and decreased *Bacteroides*. Quercetin, a member of the subclass of flavonoid polyphenols increases the abundance of *Bacteroides vulgatus* and *Akkermansia muciniphila* and concomitantly reduces *Eubacterium cylindroides* and *Bilophilia wadsworthia* to reduce the risk for diet-induced obesity which is a risk factor for CVD and hypertension. Quercetin also improves cellular energy homeostasis, fatty acid oxidation, and availability of nitric oxide by upregulating adenosine monophosphate-activated protein kinase (AMPK) expression.

Diet induced alterations in the gut microbial composition may also trigger disease states via immune activation. Regulatory T cells (Tregs) are essential immune cells to maintain immunologic self-tolerance that are categorized into two; thymus-derived and peripherally derived Tregs [46]. Importantly, SCFAs, especially butyrate, are known to induce the differentiation of peripherally derived Tregs in the colon, through G-protein coupled receptors [47]. This process is crucial to limit inflammatory activation. Furthermore, SCFAs are essential nutrients for Tregs as well as colonic epithelial cells [48]. Therefore, reduced consumption of fermentable dietary fibers may decrease colonic Treg population and predispose to chronic inflammatory states by reducing the abundance of SCFA-forming bacteria [49].

There is increasing evidence showing the link between high dietary salt and hypertension by modulation of the composition and function of the gut microbiota [50, 51]. Additional blood pressure phenotypes termed as salt sensitive and salt resistant blood pressure have an impact on the magnitude of the effect of salt on the gut microbiota. Excess dietary salt alters the gut microbiota and activates dendritic cells that in turn activates T cells and stimulate production of interleukin 17 (IL-17), tumor necrosis factor alpha (TNF-α) and interferon gamma (IFN-γ) leading to hypertension [51]. A high-salt diet may likely also disrupt the gut barrier, which results in systemic inflammation, insulin resistance, and increased blood pressure [52]. These studies indicate a link between the diet and the gut microbiota. However, more studies are needed to understand the underlying mechanisms.

### Sex differences in cardiovascular events and microbiota

Sex differences have been proposed to account for differential effects of gut microbiota and resulting metabolite effects on cardiovascular health owing to hormonal regulation and differences in dietary intake between males and female adults [39]. Sex differences in the composition of gut microbiota have been shown in both animal and human studies, although inconsistently. Using two different mice strains, Elderman et al. [53] showed that male mice had lower microbial diversity than female counterparts, with almost 12% of the variance in diversity explained by sex. Similarly, analysis of 89 different mice strains revealed significant differences in microbial composition between the sexes, although the direction of the change in each strain varied, suggesting that the influence of sex on microbiota may depend on the animal overall genotype [54].

Human studies showed similar inconsistencies. In the Human Microbiome Project Consortium males had decreased *Bacteriodes* and increased *Prevotella* populations [55], whereas in other studies, these findings were not replicated [56]. The Belgian Flemish Gut Flora Project and the Dutch LifeLines-DEEP study revealed that sex had the 10th effect size among the 69 factors shown to associate significantly with overall microbiota variation [56]. Investigation of cohorts from different ethnicities did not reveal a consistent sex-dependent microbial composition [56]. Therefore, whether sex effects on the microbiota are a determinant of the different cardiovascular risk profiles in men and women is a question awaiting answers in future studies.

### The intersection between aging and the gut microbiota in cardiovascular disease

Aging is associated with adverse cardiovascular health regardless of one’s biological sex and race [57]. The primary acquisition of microbiota occurs by exposure to the maternal one and this is heavily influenced by birth mode. The microbiota later matures, starting to resemble an adult microbiota as early as 2 years of age. During aging, there is additional exposure to external microbes through diet and contact with other environment factors such as farm animals and pets [58]. This maturation of the gut microbiota influences the development of the immune system and in some individuals it may provide an imprint for increased risk of inflammatory diseases such as inflammatory bowel syndrome, obesity, and hypertension [59]. Aging is also associated with reduction in bacterial diversity, unusual phylum proportions and decline in health promoting bacteria species [60, 61]. Specifically, the aging microbiota has been characterized by a reduction in the *Firmicutus:Bacteroidetes* ratio [62, 63] and by overpopulation of facultative anaerobes [64]. In conventionally housed mice, microbial dysbiosis, intestinal permeability, and circulating bacterial products increase with age, whereas these changes are not observed in germ-free mice, which live longer [65]. FMT from old donors is sufficient to induce phenotypes associated with aging in young recipients. For example, FMT from aged mice increases fat body mass, and food consumption, thus inducing an obesogenic phenotype in previously healthy adult mice [66]. Interestingly, metformin attenuates obesity in old mice by increasing mucin production and goblet cell mass in the gut. Metformin-induced improvement in gut health leads to decreased low-grade inflammation, a very important phenomenon seen in the elderly population that has been named inflammaging [67, 68]. Indeed, whereas healthy microbiota is associated with attenuation of markers of inflammation [64], old microbiota induces differential regulation of pathways including T cell differentiation, B-cell development, and recognition of microbes by pattern recognition receptors in young mice, further supporting a role of the gut microbiota in inflammation [69]. TMAO supplementation induces an aging-like endothelial dysfunction via reduced nitric oxide bioavailability and increased superoxide-driven oxidative stress in young mice [70]. Since TMAO and p-cresylsulfate are eliminated through the kidney [71], the age-related decline in kidney function, which is seen in both men and women, may exacerbate the systemic accumulation of these metabolites further enhancing pathways that lead to cardiovascular disease [72]. Age-associated inflammation is a risk factor for adverse cardiovascular events, thus, therapeutic approaches that target the gut microbiota may be a potential approach to promote healthy aging.

### Diagnostic and research approaches on gut microbiota state

Although the gut microbiota is too numerous to characterize, a few analytical tools are available to aid in the study of specific organisms of interest to disease. Metagenomic analysis is one of the powerful tools currently used to reconstruct microbial species and their function by examining genetic sequences. Quantification of TMAO in systemic blood is helpful in assessing CVD severity and complications. For example, elevated TMAO is present in patients with stable PAD and is a significant predictor of acute coronary syndrome, stroke, and death [7], in some cases independent of traditional risk factors [7]. In addition to TMAO, plasma levels of choline and betaine are elevated in patients with chronic heart failure. The pathogenic mechanisms of TMAO in heart failure have been previously described [11].

### Drugs and the microbiota

Certain microbial species in the gut can inactivate or lessen the potency of drugs prescribed to aid the management of CVDs. The therapeutic effects of statins are attenuated by abundant presence of *Lactobacillus, Eubacterium, Faecalibacterium*, and *Bifidobacterium* and decreased proportion of genus *Clostridium* [5], which renders these drugs relatively ineffective in decreasing LDL levels. Similarly, treatment of atrial fibrillation, atrial flutter, and heart failure using digoxin may not be efficacious when *Eggerthella lenta* strains are abundant, since they inactivate this drug [5]. Conversely, therapeutic drugs may alter the microbiota. For example, metformin, the glucose lowering drug used in diabetes mellitus treatment, cancers, CVD and other conditions increases the amount of pathogenic *Escherichia-Shigella* species [5].

### Microbiota potential therapy targets to improve cardiovascular health

We have reviewed the evidence that diet has a profound impact on microbiota composition and consequently, disease. Specific vegetarian diets that foster a good microbiota environment that is protective against CVD are recommended as the mainstay to prevent or attenuate adverse cardiovascular effects via modulation of the gut microbiota [73]. Dietary supplementation with polyphenols is quite beneficial for cardiovascular health [8].

Caloric restriction and caloric restriction mimetics are emerging as additional tools to modulate the gut microbiota and thereby promote health. Intermittent fasting and molecules such as polyphenols and beta-hydroxybutyrate decrease blood pressure by modulating the gut microbiota and attenuating inflammatory pathways [74–77]. Intermittent fasting is associated with changes in the gut microbiota including enrichment of species of the genus *Lactobacillus*, *Oscillospira*, and *Ruminococcus* and reduction of species of the genus *Akkermansia*, *Bacteroides*, and *Bifidobacterium* [78]. Most notably, the resulting gut microbial changes are associated with changes in bile acid metabolism. Microbes are responsible for modifying primary bile acids, synthesized in the liver and released into the small intestine, to form secondary bile acids [79]. Bile acids activate receptors such as the farnesoid x receptor (FXR) and TGR5 to modulate inflammation, blood pressure and vascular function [80–83]. Intermittent fasting improves availability of bile acids, which are depleted in disease states, and attenuates hypertension [84–86]. Thus, available evidence supports modulating the gut microbiota via calorie restriction modalities that improve health through mechanisms such as bile acid signaling.

Administration of probiotics (live bacteria) may offer a protection against CVDs [73]. In murine models, administration of Lactobacillus plantarum, and Lactobacillus rhamnosus GR-1 mitigated the effects of left ventricular hypertrophy, heart failure and myocardial infarction [8]. Prebiotics, nondigestible food ingredients, are known to promote bifidobacterial species and acetate-producing bacteria, thus improve gut microbiota composition and cardiovascular health [39, 44, 50]. Acetate regulates many pathways related to cardiovascular health including the upregulation of early growth response protein 1 (Egr1) transcription factor that decreases inflammation, cardiac fibrosis, and hypertrophy [7].

FMT, commonly used in treatment of *Clostridium difficile* infection and in inflammatory bowel diseases like ulcerative colitis is another emerging technique that has also been targeted to mitigate CVD [7, 8]. Evidence that FMT improves the components of the metabolic syndrome has been already provided [7]. However, the risk for introducing pathogenic microbes and toxins and increasing the risk for other pathological processes is high. An example reviewed above is that FMT from a hypertensive to a germ-free mice induces hypertension in the latter [7]. Hence, implementation of microbiota transplantation is still a challenge that needs further investigations.

Targeted therapy against gut microbiota metabolites such as TMAO may prove to be helpful as the administration of 3,3-dimethyl-1-butanol which blocks the TMA/TMAO ameliorated the harmful effects of a high-sugar and high-fat Western die on cardiac health [7].

### Clinical implications of gut microbiota and future aspects for research

It is clear that gut microbiota science has far-reaching clinical implications for management of CVDs. Diagnosis, prognosis, and monitoring of CVDs can be supplemented with characterization, quantification, and to a lesser extent, transplantation of specific gut microbiota and its metabolites. Dietary supplementation remains the safest probable method to improve the gut microbiota and its associated detrimental cardiovascular effects.

## Conclusion

Dysbiosis increases the risk for various CVDs through several mechanisms. It is associated with microbial translocation from the gut into the interstitium and perivascular tissues resulting in systemic inflammation, abnormalities of lipid and glucose metabolism, atherosclerosis, and hypertension. Western diet and timing of feeding contribute to the risk for CVD by modulating the gut microbiota. High dietary salt intake contributes to dysbiosis and development of hypertension and increases the risk for various CVDs. Sex-dependent microbial composition is emerging as one of the risk factors for CVD. However, there is still scarcity of data on this subject and it warrants further investigation. Aging is associated with a decline in health-promoting bacteria species and with enhancement of metabolic pathways that lead to cardiovascular disease. Thus, the gut microbiota is intricately involved in various CVDs. Understanding this relationship is critical for future targeted therapy to prevent and improve CVDs and to ameliorate cardiovascular adverse events. Quantification of TMAOs is an important marker for prognosis of certain cardiovascular events such as stroke, heart attack and death. Use of pre- and probiotics and TMAO inhibitors, has great potential for future therapy in managing CVDs.

### Summary

#### What is known about the topic

- The gut microbiota affects cardiovascular health. Although the mechanisms are unknown, the composition of the gut microbiota modulates risk for cardiovascular disease.
- Leaky gut is associated with inflammation. Microbial translocation elicits an inflammatory cascade that may exacerbate existing disease or induce cardiovascular diseases.

#### What this study adds

- Dysbiosis is associated with increased risk for specific diseases. Abnormal composition of the gut microbiota is linked to the pathogenesis and propagation of heart disease, atherosclerosis, hypertension, obesity, type 2 diabetes mellitus, cancer, and gastrointestinal disorders.
- High salt diet depletes lactobacillus spp. causing dysbiosis and activation of inflammatory immune response by releasing IL-17 and other inflammatory signaling molecules consequently causing blood pressure elevation.
- Gut metabolites contribute to vascular injury and thrombotic events. Choline, phosphatidylcholine, and l-carnitine found in fish, red meat, and eggs are metabolized into compounds that activate platelets and activate inflammatory pathways resulting in endothelial injury, and contributing to the progression of atherosclerosis and development of atherothrombotic events.

## Data Availability

Not applicable.
